# Melatonin Target Proteins: Too Many or Not Enough?

**DOI:** 10.3389/fendo.2019.00791

**Published:** 2019-11-15

**Authors:** Lei Liu, Nedjma Labani, Erika Cecon, Ralf Jockers

**Affiliations:** ^1^Cellular Signaling Laboratory, International Research Center for Sensory Biology and Technology of MOST, Key Laboratory of Molecular Biophysics of Ministry of Education, School of Life Science and Technology, Huazhong University of Science and Technology, Wuhan, China; ^2^Université de Paris, Institut Cochin, CNRS, INSERM, Paris, France

**Keywords:** melatonin, GPCR, QR2, ROR, PR-10, MMP-9, PEPT1/2, Glut1

## Abstract

The neurohormone N-acetyl-5-methoxytryptamine, better known as melatonin, is a tryptophan derivative with a wide range of biological effects that is present in many organisms. These effects are believed to rely either on the chemical properties of melatonin itself as scavenger of free radicals or on the binding of melatonin to protein targets. More than 15 proteins, including receptors (MT_1_, MT_2_, Mel1c, CAND2, ROR, VDR), enzymes (QR2, MMP-9, pepsin, PP2A, PR-10 proteins), pores (mtPTP), transporters (PEPT1/2, Glut1), and other proteins (HBS, CaM, tubulin, calreticuline), have been suggested to interact with melatonin at sub-nanomolar to millimolar melatonin concentrations. In this review we assemble for the first time the available information on proposed melatonin targets and discuss them in a comprehensive manner to evaluate the robustness of these findings in terms of methodology, physiological relevance, and independent replication.

## Introduction

Melatonin (N-acetyl-5-methoxytryptamine) is an evolutionary ancient molecule that is synthesized by uni- and multicellular organisms, ranging from bacteria, protists, fungi, macroalgae, plants, and animals. Melatonin has been associated with many physiological functions that evolved along the evolutionary time scale (1). Melatonin is a tryptophan derivative that due to its hydrophobicity can cross membranes by passive diffusion (2). The passive diffusion is believed to occur in pinealocytes, specialized cells of the pineal gland in vertebrates that release melatonin during the night, immediately after its circadian synthesis (3). An additional chemical property of melatonin shown *in vitro* is its antioxidant ability by scavenging free radicals (4). This antioxidant property has been proposed to be the most primitive function of melatonin being relevant along the evolutionary time scale from unicellular organisms, to plants and vertebrates. This aspect has been reviewed in another article of this series (1) and will not be addressed in this article.

Here we will focus on the mechanisms of action of melatonin, more specifically, on those effects that are mediated by its binding to molecular targets. Due to the cell-membrane penetrating properties of melatonin, extra- as well as intracellular proteins were considered as potential melatonin targets since the beginning. Over the years more than 15 different proteins have been proposed to bind melatonin ranging from receptors, enzymes, pore proteins, transporters, and various other proteins (Table 1). Examples of “functional interactions” that are often indirect, i.e., through regulation of gene transcription, including recently discussed examples such as calpain or SIRT3 will not be addressed here (38).

**Table 1:** Characteristics of melatonin target proteins.

**Protein family**	**Melatonin target**	**Affinity/Efficacy for melatonin**	**Effect of melatonin**	**Direct binding of melatonin**				**References**	**Reviews**
				**YES/NO**	**Methodology**	**Type of binding site**	**Independent replication (14)**		
Receptor	MT1	0.1 nM (Kd) (1)	Activation	YES	Ligand binding, co-crystal structure	Orthosteric (co-cyrstal, pharmacol.)	YES	(5, 6)	(7, 8)
	MT2	0.1 nM (Kd) (1)	Activation	YES	Ligand binding, co-crystal structure	Orthosteric (co-cyrstal, pharmacol.)	YES	(6, 9)	(7, 8)
	Mel1c	1 nM (Ki) (2)	Activation	YES	Ligand binding	Orthosteric (pharmacol.)	YES	(10, 11)	
	CAND2	10 nM (Ki) (2)	Activation	YES	Ligand binding	Unknown	NO	(12)	
	ROR/RZR	5 nM (Kd) (3)	activation	YES	Ligand binding	unknown	unsuccessful	(13, 14)	(15)
	VDR	20 μM (Kd) (4)	Increases affinity of Runx2 for VDR	YES	Isothermal titration calorimetry	The C-terminal ligand binding domain (LBD) of the VDR	NO	(16)	
Enzyme	QR2	1 μM (Kd) (4)	Inhibition	YES	Ligand binding, isothermal titration calorimetry, co-crystal structure	Catalytic site (co-crystal)	YES	(17, 18)	(19)
	MMP-9	50–100 μM (IC50) (5)	Inhibition	YES (12)	Docking studies, gelatin zymography assay	Catalytic site (docking)	NO	(20)	
	Pepsin	10 μM (Kd) (4)(6)	Unknown	YES	Isothermal titration calorimetry, equilibrium microdialysis	Catalytic site (docking)	NO	(21)	
	PP2A	Unknown	Suppression of PP2A inhibitor effect (11)	YES (12)	Docking studies	Near the catalyt sites (docking)	NO	(22)	(23)
Transporter	PEPT1/2	0.5–1 mM (Km) (7)	Transport of melatonin into cells and mitochondria	YES (12)	Docking studies	Substrate site (docking), competion with classical substrates	NO	(24)	
	GLUT1	Unknown	Transport of melatonin into cytoplasm, mitochondria	YES (12)	Docking studies	Substrate site (docking), competion with classical substrates	NO	(25)	(26)
	Hyp-1	Unknown (low affinity)	Binding of melatonin (11)	YES	Co-crystal strcuture	Binding site (2 sites) (co-crystal)	NO (13)	(27)	
	LLPR-10.2B	Unknown (low affinity)	Binding of melatonin (11)	YES	Co-crystal strcuture	Binding site (2 sites) (co-crystal)	NO (13)	(28)	
Others	mtPTP	0.8 μM (IC50) (8)	Inhibition of open propability	YES (12)	Electrophysiology	Unknown	NO	(29)	
	Serum albumin	10 μM (Kd) (4)(9)	Binding	YES	Ligand binding, isothermal titration calorimetry, absorption spectroscopic	Binding site	YES	(30, 31)	
	CaM	>2 mM (Kd) (10)		YES	Fluorescence spectroscopy, NMR and molecular dynamics studies (recomb. protein)	Binding site	YES	(32, 33)	
		1 nM−1 μM (IC50) (5)	Inhibition	YES (12)	Docking studies, enzyme activity	Binding site at CaM in complex with effectors	YES	(34–36)	
	Calreticuline	1 nM (Kd) (3)	Unknown	YES	Ligand binding	Unknown	NO	(37)	

*(1) [3H]-MLT saturation binding; (2) [125I]-MLT competition binding; (3) [125I]I-MLT saturation binding; (4) isothermal titration calorimetry; (5) enzyme activity inhibition; (6) Equilibrium microdialysis; (7) transport into cells; (8) inhibition of opening of mPTP; (9) Absorption spectroscopy; (10) Fluorescence spectroscopy, NMR; (11) hypothetical; (12) suggested; (13) shown for another member of the PR-10 protein family; (14) refers to the replication of the key results (binding of melatonin to targets) by another group in an independent article*.

The proposed direct targets of melatonin will be discussed regarding the half-maximal effective concentrations (EC_50_) of melatonin for the referred effects, the affinity of melatonin for the binding sites (K_d_), and the way melatonin binds to these sites, by resuming any available information on these aspects. The robustness of the proposed targets will be evaluated in terms of independent replication, a criteria that is routinely used in other fields, for example, to confirm the successful deorphanization of G protein-coupled receptors (GPCRs) (39). Finally, we will discuss how the measured EC_50_ and K_d_ values of melatonin for the different targets match with melatonin concentrations reported in different organisms, organs and biological fluids under physiological and pathological/stress conditions.

## Receptors

Receptors have been among the first suspected molecular targets of melatonin (5, 9, 10). Structural similarity of melatonin, in particular to serotonin and dopamine, and the sensitivity of melatonin-induced pigment aggregation in *Xenopus* dermal melanophores to the G_i/o_ protein inhibitor pertussis toxin pointed toward 7-transmembrane-spanning GPCRs as likely candidates for melatonin receptors (40). The cell-penetrating properties of melatonin inspired the search for additional, intracellular, melatonin receptors (Table 1).

### GPCRs

**Figure 1:**
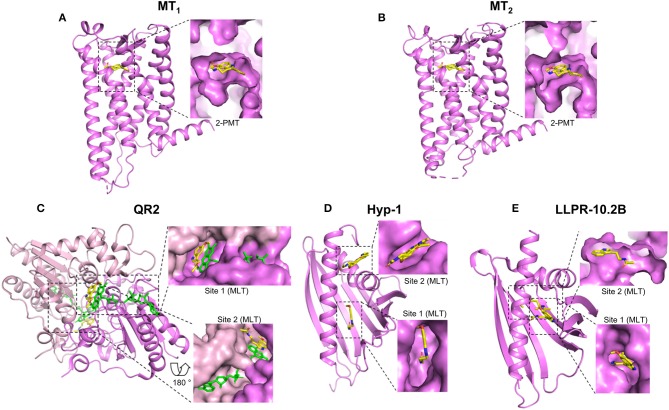
Crystal structures of melatonin target proteins in complex with melatonin or close derivatives. The full scales of the proteins are shown in cartoon in violet and the bound ligands in yellow. The ligand binding sites are highlighted by dashed rectangles and the details are shown aside by enlarged surface areas of the proteins. For those located inside, sliced views are shown to visualize the ligand. **(A)** MT_1_: MT_1_ receptor, PDB 6ME3; **(B)** MT_2_: MT_2_ receptor, PDB 6ME6; **(C)** QR2: Quinone reductase 2, PDB 2QWX, the second monomer is in light pink; FAD cofactors are shown in green **(D)** Hyp-1: St. John's wort Hyp-1 protein, PDB 5I8F; **(E)** LLPR-10.2B: Yellow lupin LLPR-10.2B protein, PDB 5MXB. MLT: Melatonin; 2-PMT: 2-phenylmelatonin. Structural views were generated using the PyMOL Molecular Graphics System (Schrodinger LLC), based on available information from the references mentioned in the text.

GPCRs are currently the best-characterized melatonin targets and are found in invertebrates and vertebrates. These receptors are classified into three groups called MT_1_ (previously Mel1a), MT_2_ (Mel1b) and GPR50 (in mammals), or Mel1c (in non-mammals) (5, 9, 10). All these receptors bind melatonin with high affinity (0.1–1 nM) (7) with the exception of GPR50, the mammalian ortholog of Mel1c that lost its ability to bind melatonin during the evolutionary divergence of the therian lineage of mammals from the monotremes (11, 41, 42). Melatonin is considered to be the natural agonist of these receptors that promotes G protein activation and beta-arrestin recruitment. These results have been replicated by many groups. Extensive pharmacological profiles have been established for these receptors with melatonin and also with various synthetic agonistic and antagonistic compounds. In addition, polymorphisms of the MT_1_ (43–45) and MT_2_ (46–49) receptors have also been identified, some of which affect the binding and signaling properties of these receptors, being factors known to influence both disease risk and/or be of pharmacogenetic relevance (8, 50). Progress on these aspects is regularly updated by the International Union of Basic and Clinical Pharmacology (IUPHAR) melatonin receptor subcommittee (7, 8, 51). Pharmacological studies have been recently complemented by crystallization studies of human MT_1_ and MT_2_ receptors co-crystallized with several melatonin analogs in their inactive states (Figures 1A,B) (52, 53). Both receptors show a high degree of amino acid homology [55% overall and 70% within the transmembrane (TM) domains], and a similar, shallow, melatonin binding pocket located within the TM domains (Figures 1A,B). The binding pose of the melatonin derivative 2-phenylmelatonin (2-PMT) proved to be very similar for both receptors with identical key residues, including the participation of the extracellular loop 2 (ECL2) (N^4.60^, F^ECL2^, Q^ECL2^, and N^6.52^) (superscripts represent Ballesteros–Weinstein nomenclature; Figures 1A,B). Interestingly, the binding pocket in the MT_1_ structure has one lateral ligand entry channel (from the membrane environment), whereas two ligand entry channels, the lateral one, and an additional one from the extracellular side, are visible in the MT_2_ structure (52–54). These different ligand entry channels as well as their different widths and differences in the overall volume of the pockets with the pocket of MT_2_ being about 50 Å^3^ larger than that of MT_1_, offer future opportunities for subtype selective drug development.

Recently, the protein product of the *CAND2* gene from *Arabidopsis thaliana* was proposed to fold into 7-transmembrane domains and to bind the radiolabeled 2-[^125^I]iodomelatonin and melatonin with high affinity (K_d_ = 0.7 nM and K_i_ ~ 10 nM, respectively) (12). This recent study has not been replicated independently for the moment. This “phytomelatonin” receptor seems to be totally unrelated to the mammalian melatonin receptors as it shows no significant overall amino acid sequence similarity (around 10%) nor does it contain any key amino acid residue known to be part of the melatonin binding pocket in melatonin receptors from animals. A pharmacological characterization of this melatonin binding site is still needed in order to compare it with mammalian melatonin receptors.

### Nuclear Receptors

Due to the cell-penetrating property of melatonin, the existence of intracellular receptors has been considered from the beginning. In this context, nuclear melatonin targets attracted much attention. This culminated in the claim that melatonin binds to and activates the retinoic acid receptor-related orphan receptor-β (RORβ), a member of the subfamily of retinoid receptors (13). However, these results could not be reproduced by other groups and were retracted later (14). In 2014, the natural ligands for ROR were identified to be sterols and oxysterols including cholesterol and its derivatives, all structurally very different from melatonin (55, 56). Despite this compelling negative evidence, it is unfortunate that numerous publications still interpret their melatonin effects by binding of melatonin to RORs and that these interpretations are repeated in many review articles. The current status on nuclear melatonin receptors has been summarized recently by Hardeland, reaching the conclusion that effects of melatonin on nuclear receptors are likely to be rather indirect (15). More recently, the Vitamin D receptor (VDR), a nuclear receptor, has been reported to bind melatonin directly with a K_d_ of 21.2 ± 1.9 μM (16). The authors found that melatonin binds to the ligand binding domain (LBD) located in the C-terminus of VDR. The binding of melatonin to VDR facilitates the interaction of VDR with the transcription factor Runt-related transcription factor 2 (Runx2), thus promoting the transcriptional activity of Runx2 indirectly. These results need to be replicated in the future but re-open clearly the long-standing discussion on the possible existence of nuclear melatonin targets.

## Enzymes

Several enzymes, like the quinone reductase 2, metalloprotease-9, pepsin, and protein phosphatase 2 have been proposed to bind melatonin directly (Table 1). There is no apparent similarity between these proteins and their proposed binding sites, and melatonin concentrations necessary for binding vary widely.

### Quinone Reductase 2 (QR2)

QR2 is most likely the best-characterized melatonin target apart from G protein-coupled melatonin receptors (19). The chase for this melatonin target started in 1988 with the identification of the MT3 (also known as ML2) melatonin binding site that was clearly different from the GPCR binding sites (17). This binding site was confirmed independently by several groups. In 2000, 5-methoxycarbonylamino-N-acetyltryptamine (MCA-NAT), a melatonin derivative with high affinity (nM) for the MT3 binding site and only modest affinity for MT_1_ and MT_2_ was used for affinity purification of MT3. The only protein retained was QR2 (57). Expression of QR2 was sufficient to replicate the pharmacology of the MT3 binding site and co-crystals showed that melatonin binds indeed to the catalytic site of QR2 (18) (Figure 1C). Using purified QR2, the K_d_ for melatonin was 1 μM in isothermal titration calorimetry (ITC) experiments and the inhibitory capacity of melatonin was in the range of 10–130 μM, depending on the functional assay used to measure QR2 activity (19). The MT3-specific pharmacological profile was recapitulated with purified QR2. The published crystal structure shows that QR2 is a symmetric dimer with two melatonin binding pockets (Figure 1C) at the interface of the two QR2 monomers (18). The poses of the two bound melatonin molecules are similar but not identical in both pockets. Hydrophobic interactions/contacts between melatonin and the protein as well as the π interactions between the FAD co-factor, such as the parallel stacking of the melatonin indole moiety on top of the FAD isoalloxazine ring and the melatonin benzene ring on top of the FAD piperazine-like moiety are important elements. QR1 belongs to the drug metabolism enzymes for which the plasticity of the catalytic site is believed to be an inherent property to accommodate natural and synthetic xenobiotic compounds from the environment. A similar broad substrate specificity can be hypothesized for QR2 which evolved from QR1 (58). QR2 has been proposed to be a membrane-associated protein although its presence as a soluble enzyme has also been claimed (19). In conclusion, although there remain some controversies about the subcellular localization of QR2, this enzyme most likely corresponds to the MT3 binding site that binds melatonin in the low μM range.

### Metalloproteinase-9 (MMP-9)

MMP-9 is located in the extracellular matrix (ECM) and contributes to ECM remodeling by cleaving various ECM components. Melatonin has been shown to negatively regulate MMP-9 expression through various mechanisms (see below). An alternative explanation has been proposed based on results obtained with purified MMP-9, which showed an inhibitory effect of melatonin in the gelatin zymography assay with an IC_50_ of 50–100 μM, suggesting a direct interaction of melatonin with MMP-9 (20). This hypothesis is compatible with molecular docking studies showing that melatonin can bind to a small cleft of MMP-9 that corresponds to its catalytic site (20). Interactions of melatonin with the key residues of the catalytic site of MMP-9 including the three zinc-coordinating histidines were suggested (20). This provides a reasonable explanation for the inhibitory effect of melatonin. MMP-9 has also been co-crystallized with other high-affinity indole-based inhibitors such as phosphinate and carboxylate derivatives (K_i_ = 10–200 nM) (59). Molecular docking studies with melatonin suggest direct binding of melatonin to MMP-9 but with lower affinity than phosphinate and carboxylate derivatives, most likely because of differences in the pose of the indole rings (20, 59). An overall protective effect of melatonin in MMP-9-dependent experimental injury models is observed in several studies including in ethanol- (60) and indomethacin-induced (61) gastric cancer, experimental colitis (62), global cerebral ischemia (63), and in Blood-Brain Barrier permeability (64). The high melatonin concentrations necessary for these effects, either in cellular models (50–1,000 μM) or *in vivo* (10–100 mg/kg), are compatible with the IC_50_ of melatonin for purified MMP-9, but raise the question of whether MMP-9 inhibition occurs under physiological conditions or whether it represents a purely experimentally-induced effect.

### Pepsin

Pepsin is a protease that is released in the stomach to catabolize ingested proteins into peptides. The active binding site of pepsin is located in a cleft between the N- and C-terminal domain with two aspartate residues, Asp32 and Asp215, which are important for its enzymatic activity. A recent biophysical study reported the direct interaction of melatonin with the catalytic site of pepsin with Asp32 being part of the suspected binging pocket (21). Binding occurred at a 1:1 stoichiometry and with a K_d_ of 10 μM as determined by titration calorimetry and equilibrium microdialysis with recombinant pepsin. Melatonin and pepsin can be both found in relatively high concentrations in the gastro-intestinal tract (see below) but their putative relationship is currently unknown. Independent replication of these results has not been reported for the moment.

### Phosphoprotein Phosphatase 2A (PP2A)

PP2A is a member of the Ser/Thr phosphatases subfamily. Inhibition or down-regulation of PP2A promotes hyperphosphorylation of neuronal proteins like Tau, followed by neuronal cell death and neurodegenerative diseases. Melatonin and its derivatives have been reported to be protective in this context (65). Several hypotheses have been put forward to explain the effect of melatonin, including the anti-oxidant activity of melatonin and a putative direct interaction of melatonin with PP2A. The latter hypothesis was fueled by the observation that gramine derivatives, which are structurally related to melatonin, suppress the inhibitory effect of okadaic acid on the enzymatic activity of PP2A (22). Docking studies indicated the possible binding of these gramine derivatives near the catalytic site of PP2A (22). A similar scenario was proposed for melatonin in a recent review article (23). Taken together, in the absence of experimental evidence for direct binding of melatonin to PP2A and for an effect of melatonin on the enzymatic activity of PP2A, this enzyme remains a hypothetical melatonin target.

## Transporters

Transport proteins help to transport molecules across membrane barriers like the plasma membrane or membranes of intracellular compartments, either passively or actively (against a concentration gradient with an energy cost). Due to its lipophilic properties, melatonin is believed to rapidly distribute all over the body through passive diffusion (2, 66). However, differences in the tissue and cellular distribution of melatonin might suggest additional regulated uptake mechanisms (26). Two proteins, the glucose transporter GLUT1 and the oligopeptide transporters PEPT1/2, have been recently proposed to transport melatonin across plasma and mitochondrial membranes (Table 1). In addition, two plant proteins, Hyp-1 and LLPR-10.2B, belonging to the pathogen-response-10 (PR-10) protein family, have been shown to bind and possibly transport melatonin (Table 1).

### Glucose Transporter 1 (GLUT1)

Along this idea of assisted transport of melatonin across membranes, Glut1 was recently proposed to be involved in cellular melatonin uptake (25). GLUT1 levels are particularly high in erythrocytes and also found in the brain, the blood-brain barrier and other tissues. Pharmacological inhibition of GLUT1 and competition of glucose uptake by high melatonin concentrations (mM), together with molecular docking studies on XylE, an *Escherichia coli* homolog of GLUT1-4 transporters, suggest that melatonin binds to GLUT1 at a site that overlaps with glucose binding (25). This interesting study will need independent replication and confirmation of direct binding of melatonin to GLUT1. A more detailed characterization of the transport capacity at melatonin concentrations below the mM range is warranted to appreciate the full physiological relevance of this proposed transport mechanism.

### Oligopeptide Transporter 1/2 (PEPT1/2)

The oligopeptide transporters PEPT1 and PEPT2 are responsible for the uptake of small peptides and peptide-like molecules in the intestine, kidney, and brain. Ectopic expression occurs also in tumors. A recent report shows that PEPT1/2 can improve the basal uptake of melatonin in cells (K_m_ = 0.5–1 mM) when applied at 50 μM concentration (24). Uptake was competed by several known PEPT1/2 substrates and docking studies suggested interaction of melatonin with key amino acid residues of the binding domain of these transporters. PEPT1/2-dependent melatonin uptake was measurable into whole cells and isolated mitochondria. PEPT1/2-dependent and PEPT1/2-independent uptakes were equally fast in reaching an equilibrium within 2–3 min, suggesting that the primary impact of PEPT1/2 would be an increase in melatonin uptake capacity of cells at micromolar to millimolar melatonin concentrations. These recent results on PEPT1/2-dependent melatonin uptake have not been replicated independently for the moment. The impact of the PEPT1/2-dependent uptake at low melatonin concentrations remains elusive. Intriguingly, expression of isoform 2 of PEPT1/2 is restricted to pinealocytes, with a pronounced circadian rhythmicity in its expression (100-fold upregulation during the dark phase), suggesting a putative role on the regulation of melatonin synthesis by a so far poorly characterized feedback mechanism (67).

### PR-10 Proteins

Plants are known to produce melatonin (68). Stress evokes a number of defense responses in plants including the expression of specific genes that encode pathogenesis-related (PR) proteins. Members of the PR-10 subclass are structurally characterized by a so-called PR-10-fold and are believed to bind small-molecule mediators, such as plant hormones. For two PR-10 proteins, Hyp-1 and LLPR-10.2B, the crystal structures in the presence of melatonin have been solved (27, 28) (Figures 1D,E). The structures of Hyp-1 and LLPR-10.2B are similar, both assemble the baseball-glove grip shape by a large seven-stranded antiparallel β-sheet over a long variable C-terminal helix, as well as the two well-defined melatonin binding sites. However, the mode of melatonin binding and the residues that participate in ligand docking are quite different in these two proteins, as the shapes of their binding cavities are not identical (Figures 1D,E). For Hyp-1, binding site 1 is located in an internal cavity of the baseball-glove grip shape and binding site 2 within the external cleft that forms around the V-shaped fork of two α-helices. Crystal structure data suggest that melatonin could have two alternative binding modes (see two melatonin molecules positioned in site 2) (Figure 1D). For LLPR-10.2B, both binding sites are within the internal cavity of the baseball-glove grip shape (Figure 1E), and the external melatonin binding site (site 1), not the deeper one (site 2), can be competed by trans-zeatin, a well-characterized PR-10 binding protein.

## Further Melatonin Targets

A range of other melatonin target proteins have been proposed and studied in more or less detail (Table 1).

### Serum Albumin

Early studies showed that melatonin and other methoxyindoles reversibly bind to a high capacity, low affinity binding site in plasma (30, 69). Fractionation studies of plasma proteins and *in vitro* studies with purified plasma proteins identified this binding site as serum albumin (30). Quantitative methods such as isothermal titration calorimetry and absorption spectroscopic with purified albumin revealed a 1:1 (melatonin: albumin) stoichiometry and a binding constant (Ka) of 1 × 10^5^ L mol^−1^ (31). Albumin fulfills thus the criteria for an efficient carrier protein with high binding capacity and low affinity to transport significant amounts of the carrier without interfering with its biological activity.

### Mitochondrial Permeability Transition Pore (mtPTP)

The mtPTP is a multi-protein complex found at the contact site between the inner and outer mitochondrial membrane. Under conditions of oxidative stress, high Ca^2+^ and low ATP levels, a number of proteins including Bax and Bad are recruited and enable the pore formation at its high conductance state, resulting in the release of Ca^2+^ into the cytosol. Recording of the mtPTP channel currents from patches of the inner mitochondrial membrane showed a concentration-dependent inhibition of mtPTP currents by melatonin (IC_50_ = 0.8 μM) (29). These electrophysiological data indicate a direct effect of melatonin on the mtPTP complex. This effect could contribute to the reported anti-apoptotic effects of melatonin, in particular under conditions of transient brain ischemia. This interesting study was not replicated nor followed up for the moment. No more information is available about the identity of the precise melatonin target candidate of the mtPTP complex, which is composed of more than 10 proteins.

### Calmodulin (CaM)

CaM is a highly conserved Ca^2+^ binding protein that regulates a large number of Ca^2+^-dependent signaling events. Studies with various biological sources containing CaM suggested high-affinity binding of melatonin to CaM (K_d_ = 0.2–1 nM) (34, 35). Subsequent fluorescence spectroscopy, NMR, and molecular dynamics studies with purified CaM confirmed the Ca^2+^-dependent binding of melatonin to CaM, but in a much lower affinity range (Kd > 2 mM) (32, 33). The interaction occurs presumably through one of the hydrophobic binding pockets of CaM, which is exposed on the protein surface upon the Ca^2+^-induced conformational changes. The huge difference (six orders of magnitude) in apparent affinity of melatonin for purified CaM vs. biological samples remains unexplained. Docking studies of melatonin to the Ca^2+^-CaM-CaM-kinaseII (CaMKII) complex suggest an improved affinity of melatonin for CaM in CaM-effector complexes (36). This is compatible with the observation that the Ca^2+^-CaM complex undergoes an additional conformational change upon interacting with CaM effector proteins. Further support for the importance of CaM effector proteins in melatonin binding to CaM comes from several studies reporting an inhibitory effect of melatonin on the enzymatic activity of CaM effectors such as phosphodiesterases (PDE) (IC_50_ ~1 nM) (70), neuronal Nitric-Oxide Synthase (nNOS) (IC_50_ ~1 μM) (71, 72) and CaMKII (IC_50_ ~10 nM) (73). The shallow concentration-response curves, spanning 5–6 orders of magnitude, suggest an indirect effect of melatonin on the enzyme activity. For nNOS, the non-competitive behavior and the fact that CaM antagonists, Ca^2+^ chelators and an excess of CaM abolish the effect of melatonin on its activity argue for CaM being the primary melatonin target protein of nNOS inhibition (72).

Binding of melatonin to microtubules has been suspected very early on (74) and subsequently characterization suggests that at nanomolar concentrations, the cytoskeletal effects of melatonin could be mediated by the Ca^2+^-CaM complex, while at higher concentrations (10 μM) “non-specific” binding of melatonin to tubulin occurs (75). These studies were not followed further and the precise nature of the melatonin target protein(s) (tubulin, CaM, other…) remains to be independently confirmed. Alternatively, signaling initiated by G protein-coupled melatonin receptors could be also responsible for the rearrangement of cytoskeleton proteins (76, 77).

In summary, despite the fact that CaM was among the first melatonin target proteins discovered, the nature of this interaction and its importance are still not clearly defined. Apparent affinities vary widely in the literature, the affinity for the purified Ca^2+^-CaM complex is low (mM range) and the interesting hypothesis of high-affinity binding of melatonin to CaM in Ca^2+^-CaM effector complexes is waiting for direct experimental validation.

### Calreticulin

Calreticulin, a ubiquitous and highly conserved Ca^2+^-binding protein that has chaperon activity and controls intracellular Ca^2+^ homeostasis, has been purified by melatonin affinity chromatography from nuclear extracts from rat hepatocytes. 2-[^125^I]iodomelatonin binding studies with recombinant GST-tagged calreticulin revealed the high-affinity binding of melatonin (K_d_ = 1 nM) that was dependent on Ca^2+^ and not competed by NAS, 4P-PDOT or luzindole, three G protein-coupled melatonin receptor ligands (37). This biochemical study qualifies calreticulin as a melatonin target candidate that merits independent replication.

## Emerging Features of Melatonin Binding to its Targets Based on Crystal Structures

Melatonin can be divided into three parts based on its chemical structure: the methoxy side chain, the middle indole ring (consisting of a benzene ring fused to a pyrrole ring) and the alkylamide side chain. Among all the published crystal structures of protein complex with melatonin or its close derivatives (Figure 1), the indole ring, in particular the benzene ring, is always involved in the melatonin-protein interaction through hydrophobic contacts or **π** interactions (Table 2). Similarly, the alkylamide side chain often interacts with target proteins (through hydrophobic contacts/hydrogen bonds), with the exception of one of the two bound melatonin molecules in QR2 (Table 2, Figure 1C). In contrast to the alkylamide side chain, the methoxy side chain does not make contacts with the target proteins in most of the cases, with the exception of the high-affinity MT_1_ and MT_2_ receptors (Figures 1A,B). This feature, together with the relatively small binding pocket and ligand entry channel, most likely define the structural basis for high-affinity binding of melatonin to these targets (52, 53). The binding preference of target proteins for the alkylamide chain might be explained by the high flexibility of this part in respect to the indole ring, thus providing several options for interactions with different proteins (“up” and “down” positions in Table 2). The short methoxy chain apparently prefers to stay within the same plane formed by the indole ring (“flat” position in Table 2) and rotate to the same side like the alkylamide chain in some cases (“down” position in Table 2).

**Table 2:** Structural elements of melatonin involved in the interactions with its target proteins.

**Receptor**	**PDB**	**Ligand**	**Affinity**	**Methoxy**		**Benzene ring**		**Pyrrole ring**		**Alkylamide**		
				**Position**	**HB**	**HC**	**Pi**	**HC**	**Pi**	**Position**	**HB**	**HC**
MT1	6ME3	2-PMT	nM	Flat	X	X				Down	X	
MT2	6ME6	2-PMT	nM	Flat	X	X				Down	X	X
QR2	2QWX	Melatonin (site 1) Melatonin (site 2)	μM	Flat Flat		X X	X X		X	Up Down		X
Hyp-1	5l8F	Melatonin (site 1) Melatonin 1 (site 2) Melatonin 2 (site 2)	mM	Down Down Flat		X X X	X X X		X	Down Down Up	X	X X
LLPR-10.2B	5MXB	Melatonin (site 1) Melatonin (site 2)	mM	Down Flat		X X		X X	X	Down Up	X X	X

*HB, Hydrogen Bonds; HC, Hydrophobic Contacts; Pi, Pi (π) Interactions*.

## Melatonin Concentrations

**Figure 2:**
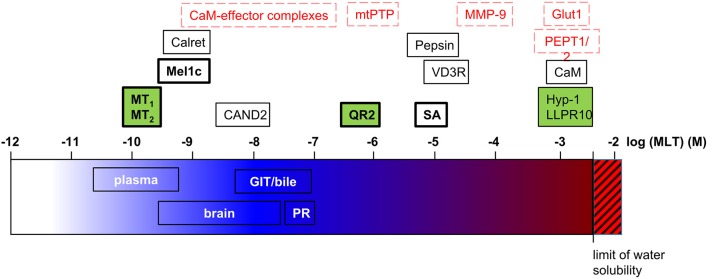
Melatonin target proteins and melatonin concentrations. Melatonin target proteins are positioned on the log scale of molar melatonin concentrations according to their affinity/efficacy for melatonin. The range of melatonin concentrations measured in biological fluids and tissues are indicated on the same scale. Legend for melatonin target proteins: black letters, demonstrated direct targets; red letters, suggested direct targets (docking, functional studies); bold letters, findings were independently replicated; green background, co-crystal structures with melatonin or phenyl-melatonin available. Calret, calreticulin; GIT, gastrointestinal tract; MLT, melatonin; PR, pineal recess; SA, serum albumin.

Taken together, more than 15 melatonin target proteins have been proposed (Figure 2) that bind melatonin at very different concentrations—from subnanomolar to millimolar concentrations. This raises the question of whether such a huge range of melatonin concentrations exists to be sensed by the different target proteins. The answer to this question is not trivial since, apart from plasma melatonin levels, there has been a lot of debate about melatonin levels in various organs and organisms, as discussed below briefly.

### In Non-vertebrates

The capacity for melatonin synthesis can be observed in all major taxa studied so far, including bacteria, dinoflagellates and other eukaryotic protists, macroalgae, plants, fungi, and various groups of invertebrate animals. Melatonin concentrations reported suggest important differences between taxa, with some studies reporting values in the upper micromolar range (26, 78). This domain suffers from a lack of data replication in the strict sense since studies are rarely performed under identical conditions, as different sources of biological material (different species, locations of collection and environmental conditions, etc.) and methods of sample preparation, melatonin extraction, and melatonin dosage are used.

In plants, more specifically in *Arabidopsis thaliana*, melatonin is believed to protect against abiotic stress through its antioxidant properties and through its action as plant hormone (68). The protein encoded by the *CAND2* gene in *Arabidopsis thaliana* has been recently shown to be a G protein-coupled receptor for melatonin that regulates stomatal closure through a H_2_O_2_ and Ca^2+^ signaling pathway (12). Interestingly, K_i_ values for melatonin are in the range of 10 nM, which are of high affinity and likely to be reached under physiological and/or stress conditions. The importance of melatonin in plants is further supported by genetic manipulation of the genes of melatonin biosynthesis as upregulation of melatonin synthesis yields improved tolerance abilities, enabling plants to better survive under hostile environmental conditions (79). The physiological relevance of melatonin binding to PR-10 proteins, the other plant proteins reported to bind melatonin, remains elusive as K_d_ values are in the mM range.

Significant melatonin levels in plants and other sources raise the question of the impact of dietary sources of melatonin (80). Many studies report melatonin content of ~1 ng/mL, however big variations are reported as well (81, 82). Several studies reported the impact of dietary melatonin from fruits on human serum melatonin levels (83), however the effects remained modest and should be also considered in light of the endogenous production of melatonin in the gastrointestinal tract (GIT) and its absence of contribution to circulating melatonin levels (see below).

### In Vertebrates

Plasma melatonin levels vary considerably between different animals and even on an individual level. In addition, melatonin levels can be altered under certain conditions, as demonstrated in humans. Indeed, there is a decline in melatonin levels with age and under several diseases, meaning that the establishment of a reference for melatonin concentration is not straightforward. In mammals including humans melatonin is produced in a circadian manner with plasma daytime melatonin levels around 5 pg/mL or less and nighttime levels rising up to 100–150 pg/mL (~0.65 nM) (84). At this concentration range, the only confirmed melatonin targets are the GPCRs, MT_1_, MT_2_, and Mel1c (Figure 2). Potential candidates are calreticulin (single study still awaiting independent confirmation) and CaM-effector complexes (still awaiting experimental validation of docking predictions). In vertebrates, the pineal gland has been identified as the primary site of rhythmic melatonin synthesis that determines plasma melatonin levels. Pineal melatonin is released in the peripheral circulation via the vein of Galen in the cerebrospinal fluid (CSF) directly via the pineal recess (PR), an evagination of the third ventricle in contact with pinealocytes. In the PR, nighttime melatonin concentrations of 20,000 pg/mL (90 nM) have been detected in sheep (85). Melatonin is then spread over the brain through the CSF from which it penetrates into the different tissues generating a melatonin gradient from the periventricular to the most distal cerebral tissues ranging from 17 to 0.35 nM at night (86). Interestingly, whereas melatonin levels vary in a circadian manner in periventricular tissues, like in the plasma, the variation was only marginal in brain tissues more distal from ventricles. The melatonin concentrations of 300 pM measured at these locations would be still sufficient to activate MT_1_ and MT_2_ receptors. Taken together, even though the highest melatonin levels close to the pineal gland are up to 100 times higher (up to 90 nM) compared to plasma levels, no obvious additional melatonin targets to the abovementioned ones have been identified so far (Figure 2). The three candidates getting the closest in terms of melatonin affinity/efficiency are QR2, VDR, and mtPTP (1–20 μM) [see (87) for review on QR2]. Since melatonin levels reported above are mean values, it cannot be excluded at present that higher melatonin levels can be reached locally, in specific sub-regions and on the subcellular level (see below). In addition, the highly diffusible nature of melatonin, being released right after its synthesis, also makes it difficult to estimate its concentration at a specific location at a given time.

Apart from the pineal gland, several other sources of melatonin synthesis have been described in vertebrates (26). The retina is the second tissue where melatonin production follows a circadian rhythm, similarly to the pinealocytes. Melatonin production from all other extrapineal sources is not rhythmic and does not contribute to the plasma levels of melatonin. Maybe the highest reported extrapineal melatonin levels are in the bile and in enterochromaffin-like cells of the GIT reaching levels that are 10–100 times higher than plasma levels (up to 50 nM) (88–91). Why these significant sources of melatonin do not contribute to circulating melatonin levels remains unclear. Considering local effects of melatonin in these organs, it can be assumed that reported melatonin levels bind a similar repertoire of potential melatonin targets as detailed above for the CSF and the brain. Whether melatonin levels measured in the GIT are sufficient to bind to pepsin (K_d_ = 10 μM) in the stomach remains to be demonstrated.

Extrapineal melatonin production has also been reported from immune cells where it occurs in an inducible manner. For example, mononuclear cells from human blood activated by zymosan or by *Escherichia coli* produce melatonin in the order of hundreds of pg/mL (~1 nM), which in turn modulates the phagocytic activity of these cells by an autocrine action mediated by melatonin receptors (92–94). Human lymphocytes, rodent peritoneal macrophages, bone marrow-derived dendritic cells and the macrophage cell line RAW 264.7 also have been reported to produce melatonin in response to diverse stimuli, including lipopolysaccharides from bacteria, serum from tumor-bearing animal models, and adrenergic stimulation (92, 95–98). Similar to what was previously mentioned about melatonin from the GIT, it is also not clear why melatonin produced by these cells does not impact the overall circulating level of melatonin.

A significant number of people take exogenous melatonin. In the USA, an estimated 3.1 million adults (1.3% of the adult population) take melatonin on a daily basis (99). Melatonin is popular for the promotion of improved sleep initiation and fast adjustment in situations of circadian misalignment (such as jet-lag when traveling over several time zones), but also as a prophylactic anti-aging treatment and as a preventive treatment for neurodegenerative diseases and cancer. Typical doses of melatonin range from 0.3 to 10 mg per day (100). At a dose of 0.3 and 2 mg of melatonin, plasma peak levels increase 2 to 3 times over endogenous peak levels, respectively (84, 101). In critically ill patients with a reduced disappearance rate of melatonin, as well as in normal healthy subjects, administration of 3 and 5 mg was reported to increase plasma peak levels 7 to 15 times reaching levels of ~50–100 nM (102, 103). It can be therefore anticipated that maximal serum peak levels of melatonin will not reach far beyond 100 nM even upon treatment with melatonin, which is ~200 times higher than endogenous peak levels and in the range of melatonin levels reported in the brain and GIT/bile. Taken together, exogenous administration of melatonin increases plasma peak level up to 200 times, but is unlikely to reach μM or mM concentrations to bind to additional, low-affinity target proteins *in vivo*.

### At the Subcellular Level

Several reports suggest that melatonin might be differentially distributed in subcellular compartments. In particular, cell nuclei and mitochondria seem to contain higher melatonin concentrations than other compartments such as the cytosol. Side-by-side comparison of melatonin and serotonin using amperometric and fluorescence measurement methods in intact cells demonstrated that extracellular melatonin, but not serotonin, equilibrates within seconds with the cytoplasm confirming that melatonin crosses biological membranes rapidly (2). Other studies suggest facilitation of melatonin transport, in particular into mitochondria, through PEPT1/2 and GLUT1 transporters (26). Recently, mitochondria isolated from neurons have been proposed to synthesize melatonin but the levels reached are unknown (104). The relative contribution of pineal melatonin synthesis to mitochondrial melatonin levels in neurons, i.e., whether melatonin is imported in or exported out of mitochondria and whether this occurs by passive diffusion or through the proposed PEPT1/2 and GLUT1 transporters, remain interesting questions to be solved in the future. The presence of melatonin in mitochondria is not only of interest because of the presumed elimination of free radicals by the antioxidant action of melatonin but also because of the presence of MT_1_ receptors in mitochondrial membranes coupled to the inhibition of cytochrome c release and apoptosis (104, 105).

Similar to mitochondria, nuclei have been proposed to contain melatonin targets. As detailed before, its nature remains to be determined in light of the inconclusive evidence for RORβ and follow-up studies on VDR will show the robustness of this recently proposed nuclear melatonin target. The presence of MT_1_ or MT_2_ receptors in the nuclear membrane cannot be completely ruled out either in analogy to other GPCRs with nuclear localization. Alternatively, calreticulin present in ER membranes could be also of relevance due to the close spatial proximity of the ER membrane and the nuclear envelop. Altogether, the subcellular distribution of locally produced melatonin and its targets are still an active and challenging object of study in the melatonin field. Progress in this field holds great promise to solve much of the mystery regarding the mismatch between melatonin concentrations required to bind to melatonin target proteins and the *in situ* concentrations measured so far.

## Conclusion

Currently 18 different melatonin targets have been proposed comprising receptors, enzymes, transporters and other proteins (Table 1). Surprisingly, 12 of them are still awaiting independent replication. The level of melatonin to which these targets respond range over 7 orders of magnitude, from sub-nanomolar to millimolar concentrations (Figure 2). Only 8 of the validated/proposed targets respond to low to moderate melatonin levels. For 5 melatonin targets, structural information is available from co-crystals. These targets provide first insights on the structural requirements for melatonin binding as they bind melatonin with high (nM), medium (μM), and low (mM) affinity concentrations. More studies will be necessary to validate the proposed targets by independent replication. Pharmacological profiles will have to be established similar to what has been done for melatonin receptors already starting back in 1975 (106). Further studies will also be necessary to determine local melatonin production and melatonin concentrations with more precision, directly at their targets in specific cellular environments and in intracellular compartments, to judge the relevance of melatonin and its targets with μM and mM affinity/efficacy. Development of non-invasive detection methods will be beneficial in this respect to capture the real levels of the highly diffusible melatonin. The authors hope that this review will provide a rational basis for a consensus of validated melatonin target proteins and help to eliminate ungrounded claims about melatonin targets, in particularly in the review literature of the melatonin field.

## Author Contributions

RJ initiated the review and wrote the first draft together with LL and EC. The initial literature research was performed by NL. All the authors participated in the editing of the manuscript and in the preparation of the figures and tables. LL generated structural models shown in Figure 1 and defined structural requirements of melatonin binding shown in Table 2.

### Conflict of Interest

The authors declare that the research was conducted in the absence of any commercial or financial relationships that could be construed as a potential conflict of interest.
